# The Association Between Poor Sleep Quality and Lipid Levels Among Dyslipidemia Patients in Thailand: A Prospective Cross-Sectional Study

**DOI:** 10.3390/healthcare13060678

**Published:** 2025-03-20

**Authors:** Jakkrit Pintacom, Suphawita Pliannuom, Nida Buawangpong, Chaisiri Angkurawaranon, Kanokporn Pinyopornpanish

**Affiliations:** 1 Department of Family Medicine, Faculty of Medicine, Chiang Mai University, Chiang Mai 50200, Thailand; aumokman1995@gmail.com (J.P.); nidalooknum@gmail.com (N.B.); chaisiri.a@cmu.ac.th (C.A.); kpinyopo@gmail.com (K.P.); 2 Global Health and Chronic Conditions Research Group, Chiang Mai University, Chiang Mai 50200, Thailand

**Keywords:** poor sleep quality, lipid levels, dyslipidemia, triglyceride

## Abstract

**Background:** Dyslipidemia increases cardiometabolic risk, but the impact of sleep quality on lipid levels remains uncertain. This study aims to assess the prevalence of poor sleep quality in patients with dyslipidemia and its association with lipid levels. **Methods:** A cross-sectional and prospective study was conducted among patients aged 20 years and older who were diagnosed with dyslipidemia at the Family Medicine Outpatient Clinic in Thailand from July to December 2023. Data were collected through interviews and a review of electronic health records. Sleep quality was assessed using the Pittsburgh Sleep Quality Index (PSQI). Blood levels of triglycerides, HDL-C, and LDL-C were measured. The association between sleep quality and lipid levels was analyzed using multivariable regression, adjusting for age, sex, body mass index, diabetes mellitus, hypertension, alcohol use, exercise, statins, fibrates, and ezetimibe. **Results:** Of the 257 participants, 183 (71.21%) were female, with a mean age of 69.31 ± 7.48 years. Overall, 86 participants (33.64%) reported poor sleep quality. A higher sum score of PSQI was significantly associated with elevated triglyceride levels (adjusted mean difference [AMD] 2.59, 95% CI 0.07–5.11, *p* = 0.044), particularly in the sleep latency domain (AMD 8.58, 95% CI 0.00–17.16, *p* = 0.050). Additionally, higher scores in the subjective sleep quality domain were significantly associated with increased LDL-C levels (AMD 8.08, 95% CI 1.70–14.60, *p* = 0.013). **Conclusions:** This study highlights a significant association between poor sleep quality and elevated triglyceride and LDL-C levels. These findings underscore the importance of integrated healthcare strategies that address both sleep quality and lipid management to mitigate cardiovascular risks.

## 1. Introduction

Dyslipidemia is a significant non-communicable disease characterized by abnormal lipid levels in the bloodstream [1], including elevated total cholesterol, low-density lipoprotein cholesterol (LDL-C), and triglycerides, as well as reduced high-density lipoprotein cholesterol (HDL-C) [1]. In a healthy state, lipid metabolism relies on the balance between HDL and LDL cholesterol. HDL, or “good cholesterol”, helps remove excess cholesterol from the bloodstream, while LDL, or “bad cholesterol”, can accumulate in blood vessels, leading to cardiovascular risks [2]. Lipid metabolism is influenced by genetic, physiological, environmental, and behavioral factors, including a sedentary lifestyle, poor nutrition, smoking, and conditions such as hypothyroidism, diabetes, and obesity. These factors contribute significantly to the development of dyslipidemia [3,4]. The global prevalence of dyslipidemia in adults ranges from 20% to 80%, depending on the population studied and diagnostic criteria used [1]. This increasing prevalence is a growing public health concern, as dyslipidemia is a key predictor of cardiometabolic risk and is associated with oxidative stress and inflammation, contributing to severe cardiovascular events and, in some cases, fatal outcomes [5,6,7,8].

Despite significant progress in understanding the risk factors for dyslipidemia, the role of sleep quality remains unclear. Sleep, like other lifestyle factors such as diet and physical activity, has been extensively studied for its impact on lipid metabolism [9]. However, evidence regarding the relationship between sleep quality and lipid abnormalities remains unclear [10]. While poor sleep quality is recognized as a determinant of overall well-being and has been linked to various chronic diseases and metabolic disorders, including dyslipidemia [11], its direct effect on lipid regulation is still ambiguous.

Several studies suggest a potential association between sleep and lipid levels. For example, good sleep quality has been associated with healthier lipid profiles, particularly among female patients [12]. Conversely, longer sleep durations have been correlated with higher triglyceride levels [13]. In Taiwan, disrupted sleep—characterized by waking up more than three times per night—was associated with elevated non-HDL cholesterol levels [14]. However, a study from the Netherlands found no significant relationship between poor sleep quality and abnormal lipid levels [10], highlighting the need for further investigation.

Poor sleep quality is common among adults seeking medical consultation [15], and has been linked to impaired cognitive function, mood disturbances, and an increased risk of neurodegenerative diseases such as Alzheimer’s disease [7]. Given its potential implications for patients with dyslipidemia, understanding sleep quality in this population and its association with lipid levels is crucial for developing comprehensive management strategies. Therefore, this study aims to assess the prevalence of poor sleep quality among patients with dyslipidemia and determine its association with lipid levels. The findings may contribute to existing knowledge and help inform future strategies for more effective dyslipidemia management by addressing sleep-related issues.

## 2. Materials and Methods

### 2.1. Study Design

This cross-sectional and prospective study was conducted among patients with dyslipidemia receiving treatment at the Family Medicine Outpatient Clinic at Maharaj Nakorn Chiang Mai Hospital, Thailand. The study was conducted from July to December 2023, was approved by the Research Ethics Committee of the Faculty of Medicine, Chiang Mai University (No. 0268, study code FAM-2566-0268), and was conducted in accordance with the Declaration of Helsinki. The study was reported in accordance with the Strengthening the Reporting of Observational Studies in Epidemiology (STROBE) statement [16].

### 2.2. Participant and Setting

The Family Medicine Outpatient Clinic at Maharaj Nakorn Chiang Mai Hospital is a primary care clinic within a university-affiliated tertiary care center in Northern Thailand. We included patients aged 20 years and older who were diagnosed with dyslipidemia, as recorded in electronic health records (EHR), with either medication or lifestyle modification, or both by convenience sampling. Patients were required to have lipid profile (total cholesterol, triglycerides, HDL-C, and LDL-C) within one month of the date when the sleep quality was collected. We excluded patients who are unable to understand the questionnaire, even with assistance from research assistants. All participants provided their consent to participate in the research before the interview session.

### 2.3. Data Sources and Contents

We collected data through interviews with participants, facilitated by research assistants and from EHR. The data collected included patient demographic data, sleep quality assessment, and data related to dyslipidemia.

Patient demographic data included age, gender, marital status, education level, income, exercise, and history of alcohol consumption and smoking. Additionally, we collected data from EHR recorded on the same date of the interview, such as weight, height, BMI, blood pressure, and comorbidities.

Sleep quality was assessed using the Pittsburgh Sleep Quality Index (PSQI) [17]. The PSQI consists of 10 questions covering seven domains: subjective sleep quality, sleep latency, sleep duration, habitual sleep efficiency, sleep disturbances, use of sleep medication, and daytime dysfunction. Each scored domain on a scale from 0 to 3. Higher scores reflect poorer sleep quality. A score of 6 or higher on the PSQI, which has a maximum possible score of 21, indicates poor sleep quality [17,18].

The data related to dyslipidemia included lipid-lowering medications (e.g., statins, fibrates, and ezetimibe) and blood lipid levels, such as triglycerides, HDL-C, and LDL-C. These data were obtained from EHR at Maharaj Nakorn Chiang Mai Hospital, disease control status regarding the 2019 ESC/EAS Guidelines for the Management of Dyslipidemia [19].

### 2.4. Statistical Analysis

Statistical analysis was conducted using Stata version 16 (Stata Corp LCC, College Station, TX, USA). To calculate the sample size, we used the formula for two independent population proportions. The proportions for poor sleep quality were 0.43 and a normal sleep quality of 0.25 in dyslipidemia patients [18]. Using an alpha error of 0.05 and a power of 0.8, the estimated sample size was 257 participants. Patient demographic data, sleep quality information, and data related to dyslipidemia are presented as numbers and proportions, mean and standard deviation (SD), or median and interquartile range (IQR) as appropriate. Chi-square tests were employed to compare across groups stratified by PSQI scores. The association between poor sleep quality and lipid levels was analyzed by using multivariable regression models and was expressed as an adjusted mean difference (AMD) with a 95% confidence interval (95% CI). Lipid levels (triglycerides, HDL-C, and LDL-C) were dependent variables. The potential confounders included age, sex, hypertension, diabetes, alcohol consumption, exercise, BMI, and lipid-lowering medication use, as illustrated in Supplementary Figure S1.

## 3. Results

**Figure 1:**
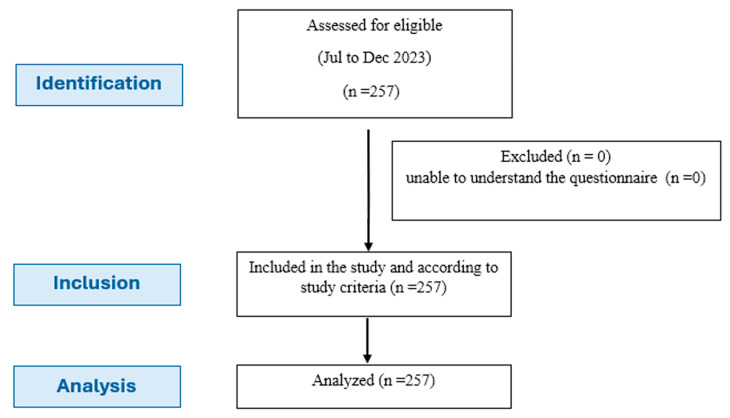
Study flow diagram.

There were 257 eligible participants, as shown in the study flow diagram (Figure 1). Out of the 257 participants, 183 (71.21%) were female, with a mean age of 69.31 ± 7.48 years. The majority of participants were receiving statin therapy (248 participants, 96.50%) and engaged in regular exercise (212 participants, 82.49%). The mean lipid levels were as follows: triglycerides, 114.67 ± 55.66 mg/dL; HDL-C, 61.92 ± 15.57 mg/dL; and LDL-C, 103.44 ± 31.39 mg/dL, as shown in Table 1. The prevalence of poor sleep quality among patients with dyslipidemia was 33.64% (86 individuals). The top three PSQI domains with the highest mean scores in the poor sleep quality group (PSQI: 6–21) were sleep duration (2.16 ± 0.09), sleep latency (1.92 ± 0.11), and subjective sleep quality (1.42 ± 0.07).

From Table 2, participants with poor sleep quality, as indicated by higher PSQI scores, were significantly associated with higher triglyceride levels (AMD 2.59, 95% CI 0.07–5.11, *p*-value 0.044) and showed a trend towards an association with lower HDL-C levels (AMD −0.63, 95% CI −1.29 to 0.04, *p*-value 0.064). Additionally, higher scores in the sleep latency domain were significantly associated with higher triglyceride levels (AMD 8.58, 95% CI 0.00 to 17.16, *p*-value 0.050). Furthermore, higher scores in the subjective sleep quality domain were significantly associated with higher LDL-C levels (AMD 8.08, 95% CI 1.70 to 14.60, *p*-value 0.013).

## 4. Discussion

In this study, we reported the relatively high prevalence of poor sleep quality among patients with dyslipidemia. Poor sleep quality, as indicated by higher PSQI scores, and abnormal sleep latency was significantly associated with elevated triglyceride level. Additionally, the subjective sleep quality domain was significantly associated with higher LDL-C levels.

Our study found that approximately one-third of patients with dyslipidemia had poor sleep quality. A previous study from China reported higher rates of poor sleep quality, with 54.5% of men and 61.2% of women with dyslipidemia experiencing poor sleep quality, based on a self-reported single-question assessment [12]. The higher prevalence in that study may be due to differences in data collection methods, particularly in how poor sleep quality was assessed. In our study, the short sleep duration domain had the highest mean score among all PSQI domains, indicating that most patients had a limited number of sleep hours. Similarly, a study in Greece found that individuals with short sleep durations had a higher prevalence of dyslipidemia (38.5%) [20]. Despite these variations, poor sleep quality remains a common issue in patients with dyslipidemia and should be addressed. However, the prevalence of poor sleep quality among patients with dyslipidemia has been less frequently studied compared to patients with diabetes mellitus or metabolic syndrome [21]. In individuals with metabolic syndrome, poor sleep quality has been reported in 40% to 60% of cases [21].

Our study found that poor sleep quality, particularly sleep latency, was significantly associated with elevated triglyceride levels, while subjective sleep quality was significantly associated with elevated LDL-C, highlighting the impact of sleep quality on lipid control. These findings align with previous research indicating that difficulties maintaining sleep and early morning awakenings were significantly associated with dyslipidemia, particularly elevated triglyceride levels [20]. Similarly, studies in other countries, including the U.S., Norway, Taiwan, Greece, and Brazil, have also reported associations between short sleep duration and sleep disturbance, affecting lipid levels, particularly by increasing triglyceride levels and decreasing HDL-C levels [10,13,14,20,22,23,24]. A study from Japan found a significant association between the sleep latency domain of the PSQI and metabolic syndrome, which has been linked to higher triglyceride levels and lower HDL-C levels [21]. Poor sleep quality may further exacerbate metabolic dysfunction and increase cardiovascular risk [21]. While some studies have suggested that short sleep duration can significantly elevate LDL-C levels [20,25], our study did not observe this association. Instead, we found a significant association between subjective sleep quality and elevated LDL-C levels. Previous research has reported varied results regarding the impact of sleep quality on LDL-C levels [26]. Notably, a study by Yeo et al. found that poor subjective sleep quality was significantly associated with elevated LDL-C, particularly in older adults and those with cardiovascular risk factors [26].

Poor sleep quality can be associated with higher triglyceride and LDL-C levels, potentially due to increased daytime fatigue and reduced physical activity, both of which can negatively impact lipid profiles [27]. Inadequate sleep can trigger acute stress responses, leading to elevated triglyceride levels [27]. Additionally, reduced physical activity due to insufficient sleep has been shown to lower HDL-C levels [20,25], further contributing to an unfavorable lipid profile, in both triglyceride and HDL-C [20]. Poor sleep quality may also lead to increased sympathetic nervous system activation, elevated cortisol levels, and chronic low-grade inflammation, which can disrupt metabolic processes and hormonal balance. These factors contribute to increased hepatic LDL-C production and reduced LDL clearance, ultimately raising overall LDL-C levels [26]. Moreover, prolonged sleep latency and difficulty maintaining sleep have been linked to a higher risk of metabolic syndrome, likely due to these disrupted metabolic processes, hormonal imbalances, and heightened stress responses [21]. Conversely, lipid metabolism may also affect sleep quality [28]. Increased visceral adipose tissue triggers inflammatory cytokine secretion, disrupting the sleep–wake cycle, while dyslipidemia-related metabolic disturbances contribute to neuroendocrine dysfunction and heightened stress responses, further impairing sleep regulation [28]. Therefore, poor sleep quality and lipid metabolism exhibit a bidirectional relationship [28].

Further studies are needed to confirm the phenomenon of why triglycerides and HDL-C, and LDL-C are associated with sleep, as well as to explore the underlying mechanisms. The association between poor sleep quality and changes in lipid levels has been a subject of significant scientific and clinical interest for a long time [29]. Poor sleep quality disrupts the body’s hormonal balance, particularly impacting cortisol, which is essential for lipid metabolism [30]. Dysregulation of cortisol due to inadequate sleep can lead to increased insulin resistance, impaired glucose metabolism, and altered lipid synthesis pathways [31]. Additionally, poor sleep patterns activate the sympathetic nervous system, increasing levels of catecholamines such as adrenaline, which can negatively affect lipid metabolism. Behavioral factors also contribute significantly; poor sleep quality is associated with changes in dietary habits, often resulting in higher consumption of unhealthy fats and sugars, which exacerbate dyslipidemia [30]. Poor sleep causes hormonal disruptions, reducing leptin (which decreases fullness) and increasing ghrelin (which heightens cravings for high-calorie foods), ultimately resulting in unhealthy dietary choices [32]. Reduced physical activity due to fatigue and decreased motivation from poor sleep further aggravates these dietary effects. Stress is another crucial factor leading to behaviors like smoking and excessive alcohol consumption that can adversely affect lipid levels [30]. However, the underlying mechanisms connecting poor sleep quality to alterations in lipid profiles are not yet fully understood. Additional research is required to clarify these relationships and determine the specific pathways involved.

### Strength and Limitation

This study has several strengths that contribute to its robustness in examining the association between sleep quality and lipid levels in patients with dyslipidemia. The use of the PSQI to assess sleep quality provides a standardized measure, enhancing the validity and comparability of the findings. Additionally, the blood lipid levels were collected during the same period as the sleep quality data, ensuring consistency in the data. Studying has some limitations. First, its cross-sectional design limits the ability to establish causality between poor sleep quality and abnormal lipid levels, only showing associations at a single point in time. Longitudinal studies are needed to explore temporal relationships and causative pathways. Second, the inclusion of participants from a single clinic and center may limit the generalizability of the findings. A larger and more diverse sample would enhance both generalizability and statistical power. Third, dietary and lifestyle factors, which can influence dyslipidemia management and serve as potential unmeasured confounders, were not fully accounted for. Future research should explore additional dietary and lifestyle factors that may affect the relationship between sleep quality and lipid levels in adults with dyslipidemia. Moreover, studies should focus on causal pathways and long-term outcomes to validate these findings further.

## 5. Conclusions

The study highlights a significant association between poor sleep quality and elevated triglyceride levels and LDL-C, emphasizing sleep quality as a modifiable factor impacting cardiovascular health through lipid profiles. These findings suggest that healthcare strategies should address both enhancing sleep quality and managing lipid levels to potentially lower cardiovascular risks associated with dyslipidemia and poor sleep. Therefore, a comprehensive treatment approach that integrates both sleep and lipid management is recommended.

## Figures and Tables

**Table 1:** Baseline characteristics among dyslipidemia patients, categorized by sleep quality (N = 257).

Variable	Total	Good Sleep Quality (PSQ-I: 0–5)	Poor Sleep Quality (PSQ-I: 6–21)	*p*-Value
	(N = 257)	(N = 171)	(N = 86)	
Age (years)	69.31 ± 7.48	69.56 ± 7.91	68.80 ± 6.56	0.444
Female (n, %)	183 (71.21)	114 (66.67)	69 (80.23)	0.023
Marital status (n, %)				0.668
Divorce	44 (17.12)	29 (16.96)	15 (17.44)	
Single	17 (6.61)	13 (7.60)	4 (4.65)	
Marriage	196 (76.26)	129 (75.44)	67 (77.91)	
Highest education level (n, %)				0.233
Primary education and lower	81 (31.52)	49 (28.65)	32 (37.21)	
Secondary education	78 (30.35)	51 (29.82)	27 (31.40)	
Bachelor’s degree and higher	98 (38.13)	71 (41.52)	27 (31.40)	
Income (n, %)				0.097
Less than 10,000 bath/month.	111 (43.19)	70 (40.94)	41 (47.67)	
10,001–30,000 bath/month	90 (35.02)	57 (33.33)	33 (38.37)	
More than 30,000 bath/month	56 (21.79)	44 (25.73)	12 (13.95)	
Comorbidities (n, %)				
Diabetes	78 (30.35)	54 (31.58)	24 (27.91)	0.546
Hypertension	191 (74.32)	122 (71.35)	69 (80.23)	0.124
Alcohol drinking (n, %)	30 (11.67)	22 (12.87)	8 (9.30)	0.401
Current smoking (n, %)	3 (1.17)	3 (1.75)	0 (0.00)	0.217
Exercise (n, %)	212 (82.49)	147 (85.96)	65 (75.58)	0.039
Duration exercise (minute/week) ^1^	140 (60, 210)	150 (60, 210)	95 (7210)	0.173
Body weight (kg)	60.04 ± 11.15	60.30 ± 11.49	59.52 ± 10.50	0.597
Body mass index (kg/m^2^)	24.71 ± 3.96	24.68 ± 4.01	24.76 ± 3.90	0.884
Blood pressure (mmHg)				
Systolic blood pressure	128.66 ± 11.13	129.13 ± 11.45	127.72 ± 10.45	0.338
Diastolic blood pressure	72.19 ± 9.36	72.19 ± 9.81	72.21 ± 8.44	0.986
Polypharmacy (n, %)	113 (43.97)	69 (40.35)	44 (51.16)	0.099
Lipid-lowering medications (n, %)				
Statin	248 (96.50)	164 (95.91)	84 (97.67)	0.467
Fibrate	2 (0.78)	2 (1.17)	0 (0.00)	0.314
Ezetimibe	15 (5.84)	9 (5.26)	6 (6.98)	0.580
Lipid levels (mg/dL)				
Triglyceride	114.67 ± 55.66	110.45 ± 51.69	123.06 ± 62.31	0.087
HDL-C	61.92 ± 15.57	62.51 ± 15.94	60.74 ± 14.81	0.392
LDL-C	103.44 ± 31.39	103.83 ± 30.93	102.66 ± 32.73	0.779
Controlled disease (n, %)	198 (77.41)	128 (74.85)	70 (81.40)	0.239
PSQI domain				
1. Subjective sleep quality	0.88 ± 0.04	0.61 ± 0.04	1.42 ± 0.07	<0.001
2. Sleep latency	1.11 ± 0.06	0.70 ± 0.05	1.92 ± 0.11	<0.001
3. Sleep duration	1.80 ± 0.04	1.62 ± 0.04	2.16 ± 0.09	<0.001
4. Habitual sleep efficiency	0.19 ± 0.05	0.02 ± 0.01	0.55 ± 0.10	<0.001
5. Sleep disturbances	1.19 ± 0.03	1.09 ± 0.03	1.138 ± 0.06	<0.001
6. Use of sleep medication	0.30 ± 0.05	0.05 ± 0.02	0.81 ± 0.13	<0.001
7. Daytime dysfunction	0.56 ± 0.04	0.36 ± 0.04	0.05 ± 0.09	<0.001
Sum score Of PSQI	5.00 ± 0.17	3.44 ± 0.09	8.18 ± 0.25	<0.001

^1^ This variable reported median and interquartile range.

**Table 2:** Association between sleep quality and lipid levels.

Lipid Levels ^1^	Triglyceride			HDL-C			LDL-C		
	AMD ^2^	95% CI	*p*-Value	AMD ^2^	95% CI	*p*-Value	AMD ^2^	95% CI	*p*-Value
1. Subjective sleep quality	−8.26	−19.97 to 3.45	0.166	0.36	−2.72 to 3.45	0.818	8.08	1.70 to 14.60	0.013 ^3^
2. Sleep latency	8.58	0.00 to 17.16	0.050 ^3^	−2.20	−4.46 to 0.06	0.056	−4.55	−9.22 to 0.12	0.056
3. Sleep duration	2.30	−9.48 to 14.08	0.701	−1.09	−4.20 to 2.01	0.489	−0.84	−7.25 to 5.58	0.797
4. Habitual sleep efficiency	0.30	−14.93 to 15.54	0.969	−0.49	−4.51 to 3.52	0.808	0.18	−8.12 to 8.48	0.966
5. Sleep disturbances	0.58	−15.54 to 16.71	0.943	−0.59	−4.84 to 3.66	0.758	−0.50	−9.28 to 8.28	0.910
6. Use of sleep medication	1.45	−7.48 to 10.37	0.750	1.38	−0.97 to 3.74	0.248	−3.53	−8.39 to 1.33	0.153
7. Daytime dysfunction	10.14	−1.01 to 21.28	0.074	−0.56	−3.50 to 2.38	0.708	−2.06	−8.13 to 4.01	0.504
Sum score of PSQI	2.59	0.07 to 5.11	0.044 ^3^	−0.63	−1.29 to 0.04	0.064	−0.64	−2.02 to 0.75	0.365

^1^ Adjusted for age, sex, hypertension, diabetes, alcohol consumption, exercise, BMI, and lipid-lowering medication use. ^2^ AMD: adjust mean difference. ^3^ A significant association is considered if *p*-value ≤ 0.05.

## Data Availability

The datasets used and/or analyzed during the current study are available from the corresponding author on reasonable request.

## Supplementary Materials

The following supporting information can be downloaded at: https://www.mdpi.com/article/10.3390/healthcare13060678/s1. Figure S1: Conceptual Figure.
