# Supplementary figures and images for: The Association Between Poor Sleep Quality and Lipid Levels Among Dyslipidemia Patients in Thailand: A Prospective Cross-Sectional Study
Source: Healthcare (Basel). 2025 Mar 20;13(6):678. doi: 10.3390/healthcare13060678 (PMC11941784; doi:10.3390/healthcare13060678)

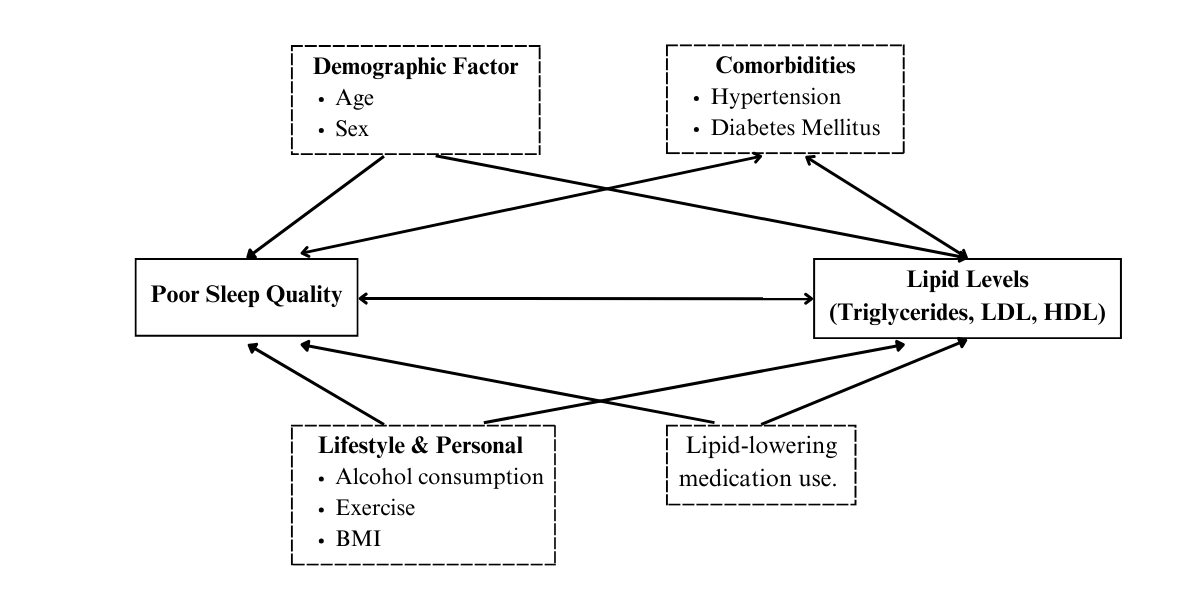

Supplement: Supplementary file 1 [file healthcare-13-00678-s001.zip › healthcare-3492221-supplementary.png]
